# Postmenopausal Osteoporosis reference genes for qPCR expression assays

**DOI:** 10.1038/s41598-019-52612-9

**Published:** 2019-11-11

**Authors:** Camilla Albertina Dantas de Lima, Suelen Cristina de Lima, Alexandre Domingues Barbosa, Paula Sandrin-Garcia, Will de Barros Pita, Jaqueline de Azevêdo Silva, Sergio Crovella

**Affiliations:** 1 Department of Genetics - Federal University of Pernambuco, Av. da Engenharia, s/n, Cidade Universitária, 50740-580 Recife, PE Brazil; 2 Laboratory of Immunopathology Keizo Asami – Federal University of Pernambuco, Av. Professor Moraes Rego, s/n, Cidade Universitária, 50670-901 Recife, PE Brazil; 3 Division of Rheumatology, Clinical Hospital, Federal University of Pernambuco, Av. Professor Moraes Rego, s/n, Cidade Universitária, 50670-901 Recife, PE Brazil; 4 Department of Antibiotics - Federal University of Pernambuco, Av. dos Economistas, s/n, Cidade Universitária, 52171-011 Recife, PE Brazil; 5 Institute for Maternal and Child Health, IRCCS Burlo Garofolo, Trieste, Italy; 6 Department of Medical, Surgical and Health Sciences, University of Trieste, Trieste, Italy

**Keywords:** Genetics, Gene expression

## Abstract

Osteoporosis (OP) is a multifactorial disease influenced by genetic factors in more than half of the cases. In spite of the efforts to clarify the relationship among genetic factors and susceptibility to develop OP, many genetic associations need to be further functionally validated. Besides, some limitations as the choice of stably expressed reference genes (RG) should be overcome to ensure the quality and reproducibility of gene expression assays. To our knowledge, a validation study for RG in OP is still missing. We compared the expression levels, using polymerase chain reaction quantitative real time (qPCR) of 10 RG (*G6PD*, *B2M*, *GUSB*, *HSP90*, *EF1A*, *RPLP0*, *GAPDH*, *ACTB*, *18 S* and *HPRT1*) to assess their suitability in OP analysis by using GeNorm, Normfinder, BestKeeper and RefFinder programs. A minimal number of two RG was recommended by GeNorm to obtain a reliable normalization. *RPLP0* and *B2M* were identified as the most stable genes in OP studies while *ACTB*, *18 S* and *HPRT1* were inadequate for normalization in our data set. Moreover, we showed the dramatic effects of suboptimal RG choice on the quantification of a target gene, highlighting the importance in the identification of the most appropriate reference gene to specific diseases. We suggest the use of *RPLP0* and *B2M* as the most stable reference genes while we do not recommend the use of the least stable reference genes *HPRT1*, *18 S* and *ACTB* in OP expression assays using PBMC as biological source. Additionally, we emphasize the importance of individualized and careful choice in software and reference genes selection.

## Introduction

Osteoporosis (OP) is a multifactorial disease, characterized by low bone mineral density (BMD) and loss of tissue microarchitecture^1–3^. The disease is influenced by genetic factors in around 50-85% of cases, especially in early or idiopathic OP^1–4^. The occurrence of fractures, the most important clinical manifestation of OP, shows heritability estimated in the range of 25–68%, presenting the highest values for younger patients and fractures that occur before the age of 70 years^1,5^. Recent Genome-Wide Association Studies (GWAS) identified several genes associated with BMD variations and fractures risk, however these associations remain to be further functionally validated^3,6,7^.

Gene expression analyses performed by polymerase chain reaction quantitative real time (qPCR) is a sensitive, accurate and commonly used method in molecular biological studies^8,9^. In 2009, in view of resolving technical challenges and to standardize qPCR experiments, the Minimum Information for Publication of Quantitative Real-Time PCR experiments (MIQE) guidelines was published^10^. One of the points highlighted by those guidelines was the need of validating reference genes for specific tissues, thereby ensuring the efficiency and quality of gene expression assays^10^. Regardless of glyceraldehyde 3-phosphate dehydrogenase (*GAPDH*) and beta-actin (*ACTB*) being traditionally used as reference genes, it is necessary to choose constitutive and stably expressed genes for different tissues and different experimental conditions^8,11^.

In spite of the efforts of Genetic Markers for Osteoporosis (GENOMOS) and Genetic Factors of Osteoporosis (GEFOS) consortiums to clarify the genetic factors involved in OP development, to our knowledge, none of the studies provided specific reference genes to be routinely used in OP gene expression assays. Therefore, we conducted an evaluation of 10 candidate reference genes commonly used in previous studies of rheumatic diseases to establish a set of specific and validated reference genes for expression analysis in OP-related genes. To this end we considered the peripheral blood mononuclear cells (PBMC), one of the main tissues analyzed in clinical-related studies in OP aiming to contribute to the advance of gene expression studies in the field of bone research.

## Results

### Specificity and amplification efficiencies

Specificity of the primers was verified by using agarose gel electrophoresis, in which a single band was detected, and the result confirmed by a single peak in the melting curve from qPCR analysis. The melting curves from all tested primers are shown in SM 1. The R^2^ and E values of the 10 candidate reference genes ranged from 0.999–1.000 and 95.12% − 104.79%, respectively (Table 1). These values were in accordance to the MIQE guidelines instructions.

**Table 1:** Primers and PCR efficiencies of the candidate reference and target genes used for OP samples.

Name	Gene	Protein function	Primer sequences/Taqman® probe reference	Product size/bp	R2	E/%
*G6PD*	Glucose-6-phosphate 1-dehydrogenase	fatty acid and nucleic acid synthesis	**F:** CCGTGATGAGAAGGTCAAGGT **R:** TACTGGCCCAGGACCACATT	72	0.999	95.12
*B2M*	Beta-2-microglobulin	small subunit of the MHC1	**F:** TGAGTGGCATGAAGAAGGTGT **R:** GGCAGTTCTTTGCCCTCTCT	77	0.999	104.79
*GUSB*	Beta-glucuronidase	degradation of dermatan and keratan sulfates	**F:** CACTGTGGCTGTCACCAAGA **R:** TCCGCATCCTCATGCTTGTT	84	1	100.51
*HSP90*	Heat shock protein HSP 90-beta	regulation of proteins in cell cycle control and signal transduction.	**F:** GCCTACTTGGTGGCAGAGAA **R:** CAGCAGAAGACTCCCAAGCA	79	0.999	98.38
*EF1A*	Elongation factor 1-alpha 1	protein synthesis	**F:** GAGGCTGCTGAGATGGGAAA **R:** CGTTCACGCTCAGCTTTCAG	74	1	102.37
*RPLP0*	60 S acidic ribosomal protein P0	ribosomal protein lateral stalk subunit P0	**F:** GCGACCTGGAAGTCCAACTA **R:** TCTGCTTGGAGCCCACATTG	100	0.999	102.02
*GAPDH*	Glyceraldehyde- 3-phosphate dehydrogenase	glycolytic enzyme	**F:** CTGATGCCCCCATGTTCGT **R:** GCAGGAGGCATTGCTGATGA	80	0.999	96.81
*ACTB*	Actin, cytoplasmic 1	cytoskeleton	Hs 99999903_m1	171	1	100
*18 S*	18 S ribosomal	ribosomal subunit	Hs 03003631_g1	187	1	100
*HPRT1*	Hypoxanthine- guanine phosphoribosyltransfe- rase	generation of purine nucleotides	**F:**ACAGGACTGAACGTCTTGCT **R:** GAGCACACAGAGGGCTACAA	74	0.99	101.43
*IFNG*	Interferon gamma	immunoregulation	**F:** TCCAAGTGATGGCTGAACTGT **R:** TCGACCTCGAAACAGCATCT	77	1	99.45

### Expression profile of candidate reference genes

**Figure 1:**
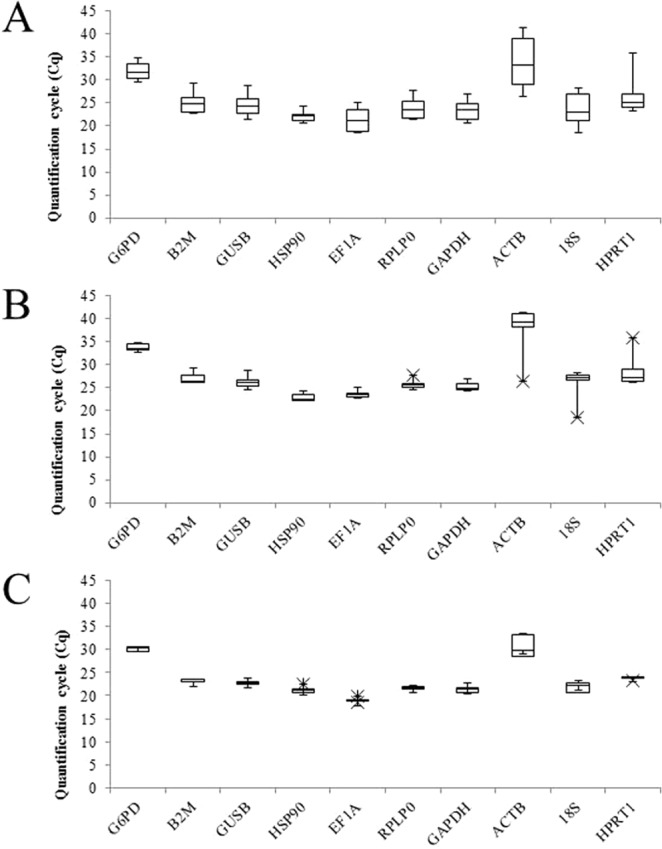
Candidate reference genes expression levels from all samples (**A**), healthy group (**B**) and patients’ group (**C**) presented as the Cq mean. The boxes show the medians values (lines across the boxes), the one-quarter (Q1) and the three-quarters (Q3) and the whisker caps indicating the minimum and maximum Cq values. The (X) represent the outliers’ values.

The quantification cycle (Cq) was used to determine the expression level of candidate genes (SM 2). Among the OP patients’ group, the *EF1A* exhibited the highest expression levels (ranging from 18.620 to 18.908 Cq values) while *HSP90* exhibited the highest expression levels in the healthy control group (ranging from 22.087 to 24.254 Cq values). In both groups, *ACTB* gene showed the lowest expression levels (ranging from 27.748 to 33.388 in patient’s group and from 26.395 to 41.410 in healthy control group). Regarding to standard deviations (SD) *G6PD* exhibited the lowest values to OP group (ranging from 0.874 to 6.233 SD values), while *HPRT1* exhibited the lowest values in healthy control group (ranging from 0.324 to 2.585) SD values. *ACTB* gene showed the highest SD values to both groups. Cq values and their variations can be appreciated in details in Fig. 1.

### GeNorm analysis

**Figure 2:**
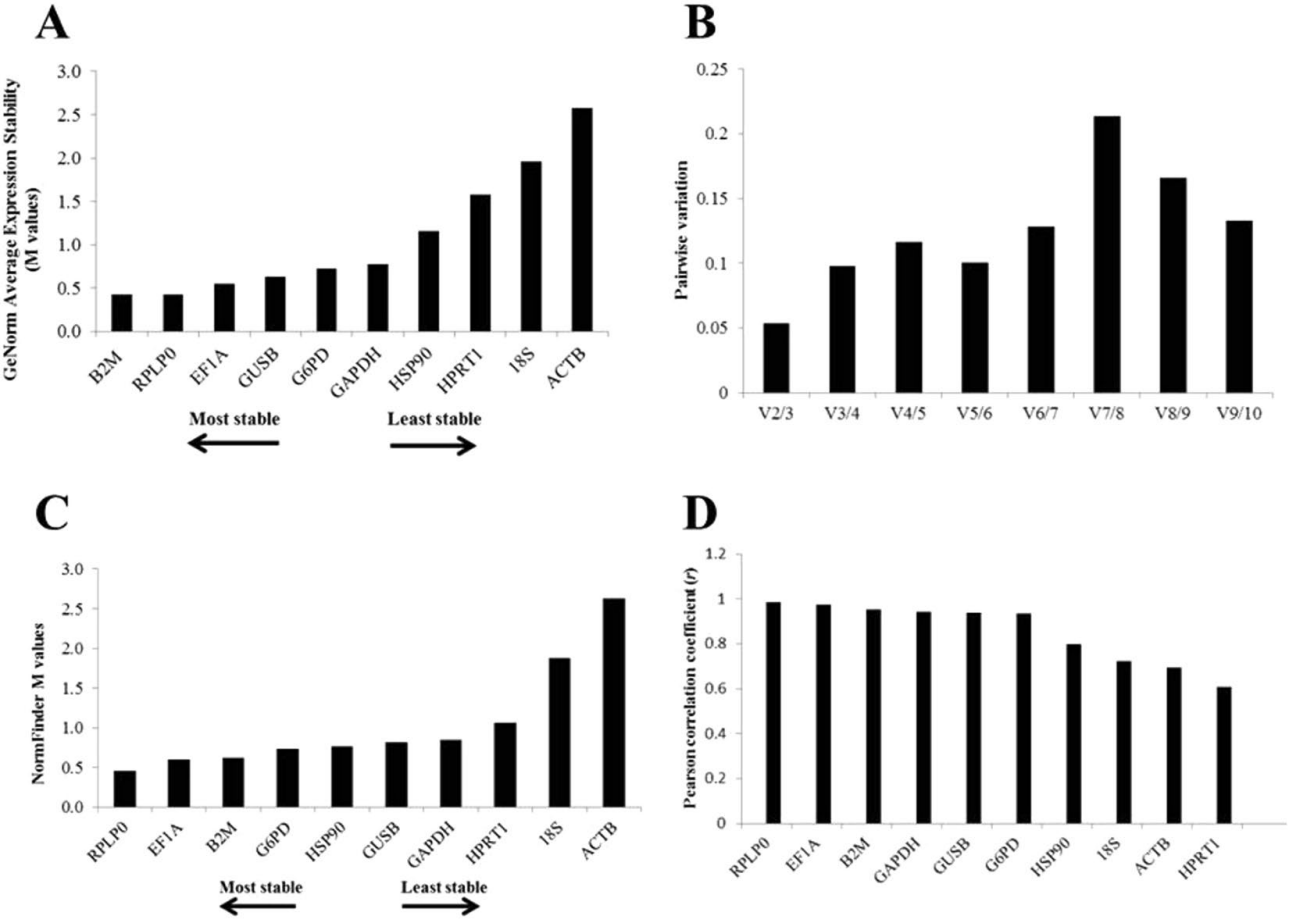
Stability analysis as revealed using different software packages. (**A**) Candidate reference gene stability analyzed using GeNorm. Low M values predict high stability while high M values indicate low stability. (**B**) Pairwise variation (Vn/Vn + 1) to determine the optimal number of reference genes required for accurate normalization by GeNorm. In this OP study, the pairwise variation value less than the cut-off (0.15) is reached with two reference genes. (**C**) Candidate reference gene stability analyzed using NormFinder. Low M values predict higher stability. (**D**) Candidate reference gene stability analyzed using BestKeeper. High Pearson correlation coefficient (*r*) predicts high stability.

In accordance to GeNorm ranking, generated from M values, *RPLP0* and *B2M* were the most stable reference genes (M = 0.423) for the present study (Fig. 2A). The pairwise variations showed V2/V3 exhibited the lowest pairwise value (0.053) (Fig. 2B). Thus, the minimal number of reference genes calculated by the algorithm and recommended to obtain a reliable normalization was two. In the present study, these genes are *RPLP0* and *B2M*. The *HPRT1*, *18 S*, and *ACTB* showed the highest M values (M = 1.579, M = 1.959, M = 2.573, respectively).

### NormFinder analysis

The NormFinder software generated a ranking, in which *RPLP0* (M = 0.460) exhibited the lowest variation values, being considered the most stably expressed gene, followed by *EF1A* (M = 0.599) (Fig. 2C). The most stable combination of genes was between both genes abovementioned (M = 0.562). The candidate reference genes showing the highest variation values were *ACTB* (M = 2.631), 18 S (M = 1.874) and *HPRT1* (M = 1.065) (Fig. 2C).

### BestKeeper analysis

Based on SD, *HSP90* presented the lowest variation value (SD [±CP] = 0.91; SD [±x-fold] = 1.87), while *ACTB* exhibited the highest variation value (SD [±CP] = 4.87; SD [±x-fold] = 29.15). Based on the correlation coefficient (*r*), the best reference gene was *RPLP0* (r = 0.987; *p* = 0.001) followed by *EF1A* (*r* = 0.975; *p* = 0.001). The lowest correlation coefficient (r) was observed to *HPRT1* (*r* = 0.606; *p* = 0.064) followed by *ACTB* (r = 0.695; *p* = 0.026) and *18 S* (*r* = 0.721; *p* = 0.019) (Fig. 2D). When both the correlation coefficient (*r)* and the SD were considered, the *HSP90* was not one of the top-ranked genes due to its lowest correlation coefficient (*r* = 0.797; p = 0.006).

### RefFinder analysis

The ranking performed by RefFinder analysis  and by the other above mentioned analyses performed in this work are shown in Table 2. According to RefFinder algorithm, *RPLP0* (GM = 1.495) followed by *B2M* (GM = 2.449) were the better reference genes for OP studies using PBMC. Similarly to other utilized analyses the *ACTB* (GM = 10), *HPRT1* (GM = 8.485) and *18 S* (GM = 8.485) were the least stable reference genes. In spite of the final ranking being similar to other studied algorithms, the individual results performed by RefFinder were substantially different from the original output provided by each program (Table 3).

**Table 2:** Reference genes rank from GeNorm, NormFinder, BestKeeper and RefFinder.

GeNorm	NormFinder	BestKeeper	RefFinder
*RPLPO/B2M*	*RPLPO*	*RPLPO*	*RPLPO*
*GUSB*	*EF1A*	*EF1A*	*B2M*
*EF1A*	*B2M*	*B2M*	*G6PD*
*G6PD*	*G6PD*	*GAPDH*	*EF1A*
*GAPDH*	*GUSB*	*GUSB*	*GUSB*
*HSP90*	*HSP90*	*G6PD*	*HSP90*
*HPRT1*	*GAPDH*	*HSP90*	*GAPDH*
*18 S*	*HPRT1*	*18 S*	*HPRT1*
*ACTB*	*18 S*	*ACTB*	*18 S*
	*ACTB*	*HPRT1*	*ACTB*

**Table 3:** Ranking Order (Better–Good–Average) showing the individual values of each software calculated by RefFinder.

Method	1	2	3	4	5	6	7	8	9	10
Delta CT	*RPLP0*	*B2M*	*GUSB*	*EF1A*	*G6PD*	*GAPDH*	*HSP90*	*18 S*	*HPRT1*	*ACTB*
BestKeeper	*HSP90*	*G6PD*	*RPLP0*	*GUSB*	*GAPDH*	*B2M*	*EF1A*	*HPRT1*	*18 S*	*ACTB*
Normfinder	*B2M*	*RPLP0*	*EF1A*	*GAPDH*	*GUSB*	*G6PD*	*HSP90*	*18 S*	*HPRT1*	*ACTB*
Genorm	*RPLP0/B2M*		*GUSB*	*EF1A*	*G6PD*	*GAPDH*	*HSP90*	*HPRT1*	*18 S*	*ACTB*
Recommended comprehensive ranking	*RPLP0*	*B2M*	*GUSB*	*G6PD*	*EF1A*	*HSP90*	*GAPDH*	*HPRT1*	*18 S*	*ACTB*

### Validation of candidate reference genes

In accordance to results from GeNorm analysis, two reference genes are enough to perform expression assays in this study. Thus, we chose the two most stable genes (*RPLP0* and *B2M*; *RPLP0* and *EF1A*) to compare with the least stable (*ACTB*, *18 S* and *HPRT1*) ones using *IFNG* expression analysis as reference. When *RPLP0* and *B2M* were used for normalization, the analysis showed that IFNG was up-regulated (4.32 fold change) in OP patients with respect to the healthy control group. When *RPLP0* and *EF1A* were used for normalization, the analysis showed that *IFNG* was up-regulated (3.53 fold change) in OP patients than healthy control group. On the other hand, when A*CTB*, *18 S* and *HPRT1* were used, the *IFNG* was up-regulated by only 2.06 fold change (FC) in OP patients with respect to the control healthy group.

**Figure 3:**
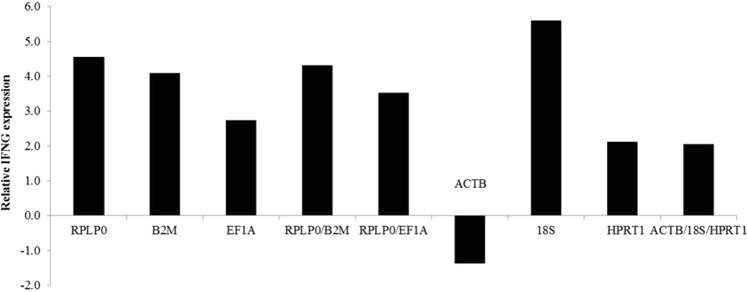
Relative quantification of *IFNG* expression using the most (*RPLP0*, *B2M* and *EF1A*) and the least (*ACTB*, *18S* and *HPRT1*) reference genes for normalization.

In the analysis of each gene independently, the group of patients presented higher expression of IFNG than healthy control group when using the following reference genes: *RPLP0* (4.56 FC), *B2M* (4.09 FC), *EF1A* (2.74 FC), *HPRT1* (2.12 FC) and *18 S* (5.61 FC). Conversely, when using either *ACTB* as reference gene individually, the expression of *IFNG* was lower in the group of patients than in control group (−1.37 FC) (Fig. 3).

## Discussion

In this study, we investigated the expression stability of the 10 most commonly used reference genes in rheumatic diseases using four statistical algorithms. To our knowledge, this is the first study to suggest the optimal reference genes for reliable expression analysis in OP using PBMC as biological source of RNA. The software used were GeNorm, NormFinder, BestKeeper and RefFinder, which are algorithms for reference gene evaluation widely accepted by the scientific community.

In spite of some differences exhibited among the software’s ranking from the four used programs, the results are relatively similar, especially in relation to the most and the least stable reference genes suggested. *RPLP0* was the most stable reference gene according to all used algorithms, followed by *B2M* (GeNorm and RefFinder) or *EF1A* (NormFinder and BestKepper). In accordance to the ranking above mentioned, the GeNorm showed a small difference in the most stable reference genes ranking in relation to NormFinder and BestKeeper. The latest two programs are recognized as able to generate more reliable data since they are less sensitive towards co-regulation and differences among the primers efficiencies^11,12^. However, in our study the relative quantitation was corrected according to primers efficiency, additionally the candidate reference genes belonged to different functional groups, which helped to correct this deficiency from GeNorm. Similarly, the RefFinder also showed in its final rank *RPLP0* and *B2M* as the best combination of reference genes. However, the calculated ranking by RefFinder were discrepant in relation to original results calculated by the programs individually, which probably occurred due to the fact that the program uses raw Cq values and did not accept corrections to reactions efficiencies (Table 3)^12^. Therefore, we do not recommend this software as a unique tool for the validation of reference genes as the output may be biased. On the other hand, the normalization factor analysis showed more similar values between *RPLP0* and *B2M* than *RPLP0* and *EF1A*. Thus, based on GeNorm output and this last analysis, we suggest the combination *RPLP0* and *B2M* as the most stable for OP gene expression studies using PBMC.

*RPLP0* encodes one out of approximately 80 ribosomal proteins in human, which are involved in protein synthesis and apoptosis processes^13^. In a previous study Ragni *et al*.^14^ classified the *RPLP0* as the most stable reference gene in expression assays performed in mesenchymal stem cell differentiation, osteoblasts precursor cells. In addition, The second most stable gene, *B2M*, encodes β2-microglobulin, which has not been cited by GWAS as a potential gene involved in OP, however, previous studies related its association with bone metabolism in tumor processes^15–17^. In spite of that, similarly to our study, Stephens *et al*.^18^ and Li *et al*.^19^ showed *B2M* as the one of the most stable reference genes in expression analysis in mouse bone cells and mesenchymal stem cells cultures, respectively. It appears that the relation between bone system and *B2M* variation is more related to cancer cells than the health bone^15,20^.

In contrast, for the low stability analysis, all the programs showed the *HPRT1*, *ACTB* and *18 S* as the three least stable reference genes. *ACTB*, as well as *GAPDH*, are used as internal controls in more than 70% of expression analyses performed by qPCR^8,21^. However, due to the very low stability in all used programs, *ACTB* was not recommended as reference gene for our analysis. *ACTB* gene encodes for β-actin, one of six different actin isoforms in vertebrates and is ubiquitously expressed in cell cytoplasm^22,23^. Actins compose the cytoskeleton, which plays critical roles in cell motility, structure, and integrity, besides acting in the regulation of gene expression^23^. Tai *et al*.^24^ showed for the first time that the factor osteo-inductive simvastatin acts in the bone regeneration by increasing actin filament organization and cell rigidity. Similarly, Elsafadi *et al*.^25^ related the involvement of distribution of the actin filament and changes in cytoskeletal organization in the osteoblastic and adipocyte differentiation of stem cells (hMSC) *in vitro*. This novel role ascribed to beta actin is a possible explanation to variation of *ACTB* gene expression in OP samples, as the disease is strictly related to the balance between bone formation and resorption.

*HPRT1* gene encodes for hypoxanthine-guanine phosphoribosyl transferase (HPRT), recognized by its transferase activity, able to catalyze purine bases guanine and hypoxanthine into their respective monophosphate nucleoside^26^. In spite of being acceptably stable in osteoblasts and osteoclasts^18^ and osteosarcoma^20^ cell culture studies, Yan *et al*.^27^ suggested that HPRT might regulate bone metabolism. The authors suggested that this protein might be involved in the development of osteoporosis through the transferase activity, which may contribute to the generation of free radical species and oxidative stress, affecting bone metabolism. Additionally, Isomura *et al*.^28^ also showed in postmenopausal rats that oxidative stress could be involved in the OP pathogenesis.

*18 S* gene encodes for ribosome 18 S rRNA subunit and, to our knowledge, there is no study reporting altered expression levels of this gene or its protein in bone cells or diseases related to changes of bone mineral density. Nevertheless, several limitations have been described so far, for instance, *18 S* expression levels being higher than the target gene^29,30^ and also the regulation of their transcription by biological and chemical agents^31^. These alterations, similarly to the other candidate genes in our analysis, are not in accordance to the criteria of constant level of the expression and the absence of influence by environmental factors required for an acceptable reference gene^30^. Besides, Ragni *et al*.^14^ and Stephens *et al*.^18^ considered the *18 S* inadequate for normalization in osteogenic and chondrogenic differentiation and mouse osteoblasts and osteoclasts analysis, respectively.

The impact of using these reference genes might be visualized in the expression levels of the target gene, which ranged greatly when different reference genes combinations were used. The *ACTB* showed that *IFNG* was down regulated in OP patients group, while according to all the other analyzed genes the *IFNG* was up regulated in the same group. In addition, when the ACTB, 18 S, and HPRT1 reference genes are used in combination, analysis showed the level of IFNG expression in the patient group (2.06 FC) was less than half the expression levels reported using the RPLP0 genes and B2M combined (4.32 FC). The expression level provided by the combination of the least unstable genes was also lower than the value obtained with the combination RPLP0 and EF1A (3.53 FC). It is also important to note that the combined relative expression (2.06 FC) changed the relative expression pattern provided by the individual genes (ACTB (−1.37 FC); 18 S (5.61 FC) and HPRT1 (2.12 FC)). The difference between the relative expression levels of IFNG when using the less stable genes in combination in relation to the individual standard of each gene, as well as the difference of the expression of these genes in relation to the more stable genes, proves the impact of the choice of the genes reference in expression analysis. These results highlight the importance in validating reference genes for specific tissues and diseases showing the aggravating effect of *ACTB* to be used in most expression analysis, which suggests that its validation has been often disregarded in expression studies^8,10^. The qPCR is a technique recognized by high sensitivity and sequence-specificity, however, the conclusions about the mRNA expression analysis may be useful only after appropriate reference gene selection^10,30^.

## Methods

### Subjects

We collected peripheral blood samples from ten postmenopausal osteoporotic women (mean age 69 ± 9.71 years old) diagnosed according WHO criteria and from ten healthy controls (mean age 63 ± 3.16 years) in postmenopausal period and without any osteometabolic disease. Individuals with cancer, inflammatory and autoimmune diseases were excluded. The present study was approved by the Research Ethics Committee of the Center for Health Sciences, Federal University of Pernambuco (CEP/CCS/UFPE n° 513/11), performed according the Declaration of Helsinki and all the participants who accepted to participate of this research provided a written informed consent.

### RNA extraction and cDNA synthesis

Total RNA was extracted using TRIzol Reagent (Invitrogen, USA) according to the manufacturer’s instructions. The RNA integrity was verified by agarose gel electrophoresis and the quantification and RNA quality was checked by Nanodrop ND 1000 spectrophotometer (Nanodrop Technologies, USA). Samples that showed integrity of 23 S and 16 S fragments and the absorbance ratio OD260/280 values from 1.8 to 2.0 were considered able to proceed to the analysis. The cDNA synthesis was performed from each RNA sample using GoScript™ Reverse Transcription System (Promega, USA) following the manufacturer’s instructions.

### Selection of candidate reference genes and PCR primer design

The selection of candidate reference genes was performed by employing the most used reference genes from researchers in humans^32,33^. Besides, reference genes commonly used in rheumatic diseases were added to the analysis. A total of 10 genes were selected: glucose-6-phosphate dehydrogenase (*G6PD*), β2-microglobulin (*B2M*), β-glucuronidase (*GUSB*), heat shock protein 90 (*HSP90*), elongation factor 1 alpha (*EF1A*), ribosomal protein P0 (*RPLP0*), *GAPDH*, *ACTB*, *18 S* ribosomal RNA (18 S) and hypoxanthine phosphoribosyltransferase 1 (*HPRT1*). All gene sequences were obtained from GenBank (www.ncbi.nlm.nih.gov/genbank/) and all primers were designed by NCBI/Primer-BLAST (www.ncbi.nlm.nih.gov/tools/primer-blast/). For *ACTB* and *18 S* we used Taqman probes. Sequence details are shown in Table 1.

### Amplification efficiency testing

The efficiency value for each primer (E) was determined by slopes of standard curves from five 10-fold serial dilution points for each cDNA sample, starting with 5 ng of cDNA. The acceptable values were defined between 95% and 105%. For *ACTB* and *18 S* (Taqman probes), we considered a reaction efficiency of 100% ensured by manufactures’ information.

### Quantitative real-time PCR assay

The qPCR was performed on the ABI 7500 (Applied Biosystems, USA) platform using 5 ng of cDNA, 10 µM of each primer, 5 μL SYBR Green Master Mix (1×) (Thermo Fischer Scientific, USA) and ultrapure water to a final volume of 10 μL. The Taqman assays were performed following the manufactures’ instructions. The melting curve was analyzed to confirm the specificities of the amplification reactions.

### Data analysis of gene expression stability

Three statistical algorithms from Excel based free software packages were used to evaluate the expression stability of 10 candidate reference genes to OP: GeNorm (Version v3.5)^34^, NormFinder (Version 20)^35^ and BestKeeper (Version 1)^36^. Additionally, the web-based online tool RefFinder^37^ and GeNorm were used to assist in the ranking and to calculate the optimal combination and minimal number of the candidate reference genes, respectively.

### GeNorm

The GeNorm uses the geometric means to determine gene expression normalization factor and stability value (M) for each gene^34^. M values less than 1.5 are acceptable and the lowest values are considered most stable. Then, the software performs a pairwise comparison (Vn/n + 1) adding genes, one by one, in order to set the most stable reference genes until a cut-off less than 0.15 for determining the minimal number of the candidate reference genes^8,11,34,38^.

### NormFinder

The NormFinder uses the standard curve or the delta-Ct method from the transformation of Ct (cycle threshold) values in a linear scale^35^. The program estimates the expression variation from candidate genes, providing a stability value (M) for each gene, and the variation between sample subgroups^35^. Similar to GeNorm, the lowest M values are considered most stable^11,12,35^.

### BestKeeper

The BestKeeper uses the geometric mean from candidate reference genes and the software provides a correlation coefficient (*r*) of each gene, besides to calculate standard deviation (SD) and coefficient of variation (CV) from the samples Ct-values^36^. It is recommended SD to be [±CP] < 1, SD [±x-fold] < 2 and correlation coefficient to be as higher as possible, close to the value 1^36,38^. Low SD and CV associated to high correlation coefficient (*r*) values indicate a stable reference gene, however the program does not perform a ranking order from the analyzed genes^11,36^. De Spiegelaere *et al*.^12^ highlighted that correlation coefficient (*r*) is a better parameter to assess the most stable genes than the standard deviation because the first one is able to analyze the correlation of each gene with the BestKeeper Index from the geometric mean from the studied reference genes. Thus we chose this measurement to evaluate and to perform the rank of reference genes in BestKeeper analysis.

### RefFinder

The RefFinder is a web-based platform (http://fulxie.0fees.us/?type=reference) which integrates the three software packages abovementioned and additionally performs the comparative ∆∆Ct method^12,37,39^. The RefFinder only uses Cq (cycle quantification) to perform the reference genes rank through of geometric mean values (GM), without any possibility to include PCR efficiency^12^. Therefore, the RefFinder platform was used as complementary tool to assess reference gene stability.

### Reference and target genes analysis

After the determination of optimal combination and minimal number of the candidate reference genes by software analysis, the most stable combination and the recommended candidate reference genes were used to perform the normalization from geometric media. To validate these potential reference genes, the IFNG was used as target gene (Table 1, SM 1). The selection of IFNG is due to the fact this cytokine is an immune-derived cytokine, with active roles in differentiation of osteoclasts and osteoblasts^40^.

## Conclusions

According to our results, we recommend the use of *RPLP0* and *B2M* as the most stable reference genes to OP studies, as we showed their lower variation impact and influence on the evaluation of a target gene expression. On the other hand, we do not recommend the use of the least stable reference genes (*HPRT1*, *18 S* and *ACTB*) in OP expression assays. Additionally, we emphasize a carefully choice of software packages to be used for reference gene selection. Finally, we highlighted that future studies should check the stability of the reference genes as other experimental methods and backgrounds of patients such as age and different cell sources. We suggest that, based on the present study, a smaller set of candidate reference genes herein showed may be used for similar analyses.

## Supplementary information

Supplementary Information

## References

[1] Rocha-Braz MGM, Ferraz-de-Souza B (2016). Genetics of osteoporosis: searching for candidate genes for bone fragility. Arch. Endocrinol. Metab..

[2] Riancho J, Hernández J (2012). Pharmacogenomics of osteoporosis: a pathway approach. Pharmacogenomics.

[3] Karasik D, Rivadeneira F, Johnson ML (2016). The genetics of bone mass and susceptibility to bone diseases. Nat. Rev. Rheumatol..

[4] Yao S (2017). Regulatory element-based prediction identifies new susceptibility regulatory variants for osteoporosis. Hum. Genet..

[5] Mäkitie RE, Kämpe AJ, Taylan F, Mäkitie O (2017). Recent Discoveries in Monogenic Disorders of Childhood Bone Fragility. Curr. Osteoporos. Rep..

[6] Li WF (2010). Genetics of osteoporosis: Accelerating pace in gene identification and validation. Hum. Genet..

[7] Mencej S, Albagha OME, Prezelj J, Kocjan T, Marc J (2008). Tumour necrosis factor superfamily member 11 gene promoter polymorphisms modulate promoter activity and influence bone mineral density in postmenopausal women with osteoporosis. J. Mol. Endocrinol..

[8] Wang H (2015). Tissue-specific selection of optimal reference genes for expression analysis of anti-cancer drug-related genes in tumor samples using quantitative real-time RT-PCR. Exp. Mol. Pathol..

[9] Wang P (2016). Selection of Suitable Reference Genes for RT-qPCR Normalization under Abiotic Stresses and Hormone Stimulation in Persimmon (Diospyros kaki Thunb). PLoS One.

[10] Bustin SA (2009). The MIQE Guidelines: Minimum Information for Publication of Quantitative Real-Time PCR Experiments. Clin. Chem..

[11] Normann KR (2016). Selection and validation of reliable reference genes for RT-qPCR analysis in a large cohort of pituitary adenomas. Mol. Cell. Endocrinol..

[12] De Spiegelaere W (2015). Reference gene validation for RT-qPCR, a note on different available software packages. PLoS One.

[13] Artero-Castro A (2011). Expression of the ribosomal proteins Rplp0, Rplp1, and Rplp2 in gynecologic tumors. Hum. Pathol..

[14] Ragni E, Viganò M, Rebulla P, Giordano R, Lazzari L (2013). What is beyond a qRT-PCR study on mesenchymal stem cell differentiation properties: How to choose the most reliable housekeeping genes. J. Cell. Mol. Med..

[15] Josson S (2011). Β2-Microglobulin Induces Epithelial To Mesenchymal Transition and Confers Cancer Lethality and Bone Metastasis in Human Cancer Cells. Cancer Res..

[16] Hsu YH, Kiel DP (2012). Genome-wide association studies of skeletal phenotypes: What we have learned and where we are headed. J. Clin. Endocrinol. Metab..

[17] Levy R, Mott RF, Iraqi FA, Gabet Y (2015). Collaborative cross mice in a genetic association study reveal new candidate genes for bone microarchitecture. BMC Genomics.

[18] Stephens AS (2011). Internal control genes for quantitative RT-PCR expression analysis in mouse osteoblasts, osteoclasts and macrophages. BMC Res. Notes.

[19] LI XIUYING, YANG QIWEI, BAI JINPING, XUAN YALI, WANG YIMIN (2015). Evaluation of eight reference genes for quantitative polymerase chain reaction analysis in human T lymphocytes co-cultured with mesenchymal stem cells. Molecular Medicine Reports.

[20] Rienzo M (2013). Identification of valid reference housekeeping genes for gene expression analysis in tumor neovascularization studies. Clin. Transl. Oncol..

[21] Klenke Stefanie, Renckhoff Kristina, Engler Andrea, Peters Jürgen, Frey Ulrich H. (2016). Easy-to-use strategy for reference gene selection in quantitative real-time PCR experiments. Naunyn-Schmiedeberg's Archives of Pharmacology.

[22] Rubenstein PA (1990). The functional importance of multiple actin isoforms. BioEssays.

[23] Bunnell TM, Burbach BJ, Shimizu Y, Ervasti JM (2011). β-Actin specifically controls cell growth, migration, and the G-actin pool. Mol. Biol. Cell.

[24] Tai IC (2015). Simvastatin enhances Rho/actin/cell rigidity pathway contributing to mesenchymal stem cells’ osteogenic differentiation. Int. J. Nanomedicine.

[25] Elsafadi M (2016). Transgelin is a TGFβ-inducible gene that regulates osteoblastic and adipogenic differentiation of human skeletal stem cells through actin cytoskeleston organization. Cell Death Dis..

[26] Kelley, R. E. & Andersson, H. C. *Disorders of purines and pyrimidines*. *Handbook of Clinical Neurology* **120**, (Elsevier B.V., 2014). 10.1016/B978-0-7020-4087-0.00055-3 24365355

[27] Yan, B., Li, J. & Zhang, L. Identification of B cells participated in the mechanism of postmenopausal women osteoporosis using microarray analysis. **8**, 1027–1034 (2015). PMC4358544 25785089

[28] Isomura H (2004). Bone metabolism and oxidative stress in postmenopausal rats with iron overload. Toxicology.

[29] Paolacci AR, Tanzarella OA, Porceddu E, Ciaffi M (2009). Identification and validation of reference genes for quantitative RT-PCR normalization in wheat. BMC Mol. Biol..

[30] Kozera B, Rapacz M (2013). Reference genes in real-time PCR. J. Appl. Genet..

[31] Nicot N, Hausman JF, Hoffmann L, Evers D (2005). Housekeeping gene selection for real-time RT-PCR normalization in potato during biotic and abiotic stress. J. Exp. Bot..

[32] Applied Biosystems (2008). Guide to Performing Relative Quantitation of Gene Expression Using Real-Time Quantitative PCR. Gene Expression.

[33] Roche Applied Science. Human Reference Gene Panel, 384. **1**, 5–6 (2009).

[34] Vandesompele J (2002). Accurate normalization of real-time quantitative RT-PCR data by geometric averaging of multiple internal control genes. Genome Biol..

[35] Andersen CL, Jensen JL, Ørntoft TF (2004). Normalization of real-time quantitative reverse transcription-PCR data: A model-based variance estimation approach to identify genes suited for normalization, applied to bladder and colon cancer data sets. Cancer Res..

[36] Pfaffl MW, Tichopad A, Prgomet C, Neuvians TP (2004). Determination of stable housekeeping genes, differentially regulated target genes and sample integrity: BestKeeper - Excel-based tool using pair-wise correlations. Biotechnol. Lett..

[37] Xie F, Xiao P, Chen D, Xu L, Zhang B (2012). miRDeepFinder: A miRNA analysis tool for deep sequencing of plant small RNAs. Plant Mol. Biol..

[38] Liu Deshui, Shi Lindan, Han Chenggui, Yu Jialin, Li Dawei, Zhang Yongliang (2012). Validation of Reference Genes for Gene Expression Studies in Virus-Infected Nicotiana benthamiana Using Quantitative Real-Time PCR. PLoS ONE.

[39] Ma Q, Zhuang Z, Feng W, Liu S, Tang Q (2015). Evaluation of reference genes for quantitative real-time PCR analysis of gene expression during early development processes of the tongue sole (Cynoglossus semilaevis). Acta Oceanol. Sin..

[40] Tang, M., Tian, L., Luo, G. & Yu, X. Interferon-gamma-mediated osteoimmunology. *Front. Immunol*. **9** (2018). 10.3389/fimmu.2018.01508 PMC6033972 30008722

[41] Lima, C. A. D. Análise do perfil genético e funcional das citocinas IL-23, IL-17, IL-12 e IFN-γ e suas relações com a osteoporose primária pós-menopausa. (Federal University of Pernambuco, 2017).

