# Supplementary material for: Postmenopausal Osteoporosis reference genes for qPCR expression assays
Source: Sci Rep. 2019 Nov 11;9:16533. doi: 10.1038/s41598-019-52612-9 (PMC6848520; doi:10.1038/s41598-019-52612-9)
Supplement: Supplementary file 1 — Supplementary Information [file 41598_2019_52612_MOESM1_ESM.pdf]

Postmenopausal Osteoporosis reference genes for qPCR expression assays

Camilla Albertina Dantas de Lima[1,2]; Suelen Cristina de Lima[2]; Alexandre Domingues Barbosa[2,3]; Paula Sandrin-Garcia, P[1,2]; Will de Barros Pita[4]; Jaqueline de Azevêdo Silva[1,2*§] and Sergio Crovella[5,6§]

1 – Department of Genetics - Federal University of Pernambuco, Av. da Engenharia, s/n, Cidade Universitária, 50740-580, Recife - PE, Brazil.

2 – Laboratory of Immunopathology Keizo Asami – Federal University of Pernambuco, Av. Professor Moraes Rego, s/n, Cidade Universitária, 50670-901, Recife - PE, Brazil.

3 - Division of Rheumatology, Clinical Hospital, Federal University of Pernambuco, Av. Professor Moraes Rego, s/n, Cidade Universitária, 50670-901, Recife - PE, Brazil.

4 – Department of Antibiotics - Federal University of Pernambuco, Av. dos Economistas, s/n, Cidade Universitária, 52171-011, Recife - PE, Brazil.

5 – Institute for Maternal and Child Health, IRCCS Burlo Garofolo , Trieste , Italy.

6 –Department of Medical, Surgical and Health Sciences, University of Trieste, Trieste , Italy.

***Corresponding Author: Jaqueline.azevedo@ufpe.br**

[§]*SC and JAS equally contributed to this manuscript.*

ORCID: 0000-0003-0320-3674

Phone number: +55 81 21267220

Short Title: Reference Genes for Osteoporosis studies

Supplementary material

SM.1 Melting curves of the candidate reference and target gene. The melting curves of the ACTB and 18S genes are not shown given that these genes were studied by Taqman probes.

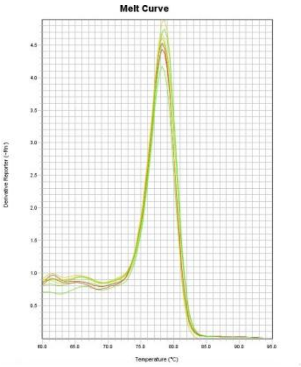

G6PD

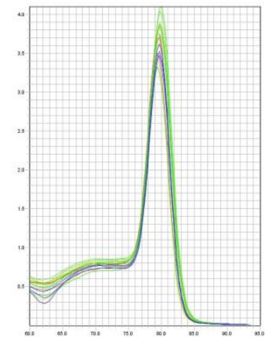

B2M

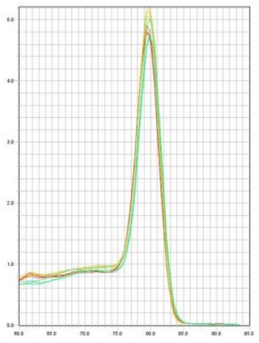

GUSB

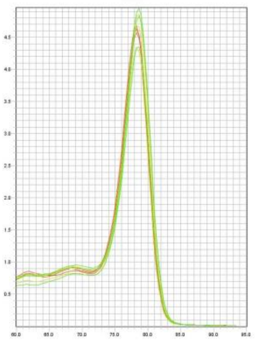

HSP90AB1

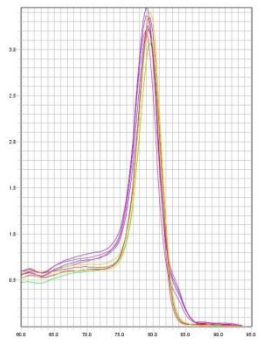

EF1A

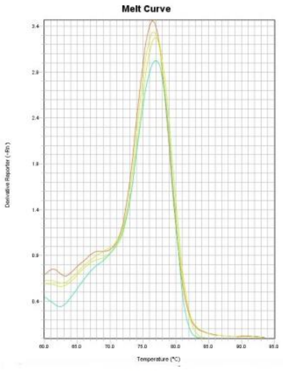

RPLP0

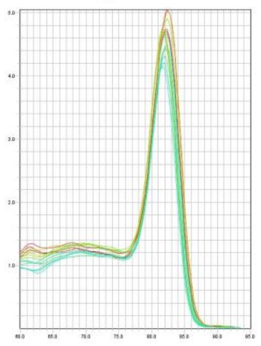

GAPDH

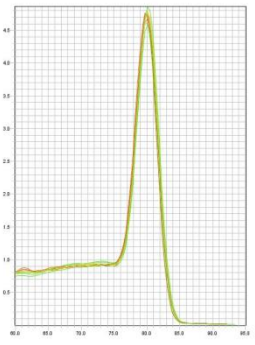

HPRT1

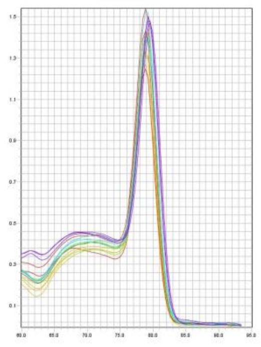

IFNG

SM 2. Cq values means of the candidate reference genes expressed in OP patients and healthy group.

	G6PD	*B2M*	*GUSB*	*HSP90*	*EF1A*	*RPLP0*	*GAPDH*	*ACTB*	*18S*	*HPRT1*
OP Patients	30.110	23.160	22.628	21.246	18.991	21.769	21.361	30.567	21.877	23.798
Healthy Control	33.741	27.142	26.305	22.871	23.639	25.732	25.320	37.285	25.656	28.868
